# Wood’s Operations for Radical Cure of Hernia

**Published:** 1868

**Authors:** Benjamin Howard

**Affiliations:** Lecturer on Operative and Minor Surgery in the Medical College of the University of New York


### ARTICLE VII.

WOOD'S OPERATION FOR RADICAL CURE OF HERNIA. By BENJAMIN HOWARD, M. D., Lecturer on Operative and Minor Surgery in the Medical College of the University of New York.

From successive observations of the progress of several of Mr. Wood's patients, I was induced to avail myself of the first opportunity of performing his operation for the radical cure of hernia.

The following observations are chiefly concerning the manner of prosecuting one only of the several steps of the operation. The inferences respecting the general value of the operation, if indeed such could be allowed from what is here exhibited, are, I think, clearly favorable.

July 18, 1866, I performed this operation upon Timothy Sullivan, a healthy laborer, aged 25, for an oblique inguinal hernia of the left side, its first occurrence dating back about two years prior to the operation.

Although the hernia was not large, its inconvenience was such as to render the patient anxious for the operation. With the assistance of Dr. Hackley, of the New York City Hospital, and others, the patient was etherized and the operation performed; the needle and wire, with the use of which I had become somewhat familiar through the frequent kindness of its author, were bought from the maker employed by himself. No obstacle was encountered in the steps of the operation; but when about transfixing the fascia in front of the cord, I felt a little undecided which of the two methods of doing it described by Mr. Wood to adopt. The first is as follows: "The sac of the hernia and the fascia covering it opposite the scrotal aperture is then pinched up between the finger and thumb, and the spermatic cord is slipped back from their grasp, from without inward and a little upward in the direction of the incision across the scrotum, close to and in front of the spermatic cord; a slight twist given to the point of the needle will enable it to take up all the structures which lie in front of the spermatic cord, and at the same time to enter and emerge entirely within the limits of the scrotal incision."

The other method is: "In still smaller cases, wherein the sac does not descend much beyond the external ring, the last step of transfixing the fascia must be performed nearer to the insertion of the pillars of the ring. In accomplishing this manœuvre, the needle may also be made to take up a portion of the pillars themselves close to their respective insertions on the inside of the spermatic cord. The crest of the pubis will afford a good guide and protection to the deeper parts, the point of the needle being made to slide close to the bone."

As this case corresponded to the class last mentioned, the transfixion was effected as therein directed, care being taken to make the point of the needle "slide close to the bone."

The wire was now in situ, crossing itself and transfixing the parts as the operation requires, the free dependent ends were twisted upon each other, and all was ready for permanent invagination.

It was here I encountered the difficulty I had feared might arise. The fixation of the parts effected by making the "wire slide close to the bone" and "take up also a portion of the pillars themselves close to their insertions," arrested the progress of invagination, making its completion impossible, and the last attachment of the wire could not be drawn up into the canal so as to plug it throughout its course.

This is the point to which I would call the attention of those contemplating this operation; and, in order that they may be spared the same difficulty encountered by myself, I would advise the adoption of the first of the two methods above described, in transfixing the sac or the scrotal tissue in front of the cord.

Though strongly inclined to withdraw them, the wires were left as placed. The operation was completed, and the wound dressed in the usual manner.

Between the second and fourth days there was more local inflammation and general disturbance than I had observed in any of Mr. Wood's cases in King's College Hospital. This was doubtless largely owing, however, to the circumstances; the patient being confined in a small apartment having a temperature at midday of 102 deg. Fah. for several days in succession, and swarming with flies and mosquitoes.

As the patient insisted that the presence of the wires was the sole source of all his discomfort, I yielded to his importunity and removed them on the fourth, instead of about the tenth day after the operation.

I regretted this circumstance very much, fearing it would deprive this, the first operation of its kind in the city, of its chief advantages. In this regard, however, the results disappointed my apprehensions.

After removal of the wire, there was considerable suppuration and tenderness, both of which daily diminished, so that at the end of two weeks the patient began to come to my office, the wound was nearly healed his general health and spirits were good, and he felt great confidence in the success of the operation.

He called again six weeks after the operation, and stated that he had been at work at *coal-heaving.* I tested the parts thoroughly, but could procure no sign of impulse; the course of the canal felt as if occupied throughout by a hard cord.

September 21 I again saw the patient, when he expressed great gratitude for what he regarded as a sound and radical cure.

The patient soon after left the city, since which time I have been unable to obtain further information about the case.

Although what I have described as an impediment in the operation did not prevent complete success as far as the history of the case is known, I deem it worth while to state these particulars, as I apprehend the same difficulties may occur to others, tending to embarrass, if not to discourage them in the prosecution of this very rational and promising mode of treatment. To those who are inexperienced in this operation, then, I suggest the selection of the first of the two methods described as above for transfixion of the scrotal fascia, or the sac, in front of the cord, because thereby *the invagination is more easy and complete, the operation more satisfactory, and the cure is likely to be more sound.*

